# Expression of Inducible Heat Shock Proteins Hsp27 and Hsp70 in the Visual Pathway of Rats Subjected to Various Models of Retinal Ganglion Cell Injury

**DOI:** 10.1371/journal.pone.0114838

**Published:** 2014-12-23

**Authors:** Glyn Chidlow, John P. M. Wood, Robert J. Casson

**Affiliations:** 1 Ophthalmic Research Laboratories, South Australian Institute of Ophthalmology, Hanson Institute Centre for Neurological Diseases, Frome Road, Adelaide SA-5000, Australia; 2 Department of Ophthalmology and Visual Sciences, University of Adelaide, Frome Road, Adelaide SA-5000, Australia; Instituto Murciano de Investigación Biosanitaria-Virgen de la Arrixaca, Spain

## Abstract

Inducible heat shock proteins (Hsps) are upregulated in the central nervous system in response to a wide variety of injuries. Surprisingly, however, no coherent picture has emerged regarding the magnitude, duration and cellular distribution of inducible Hsps in the visual system following injury to retinal ganglion cells (RGCs). The current study sought, therefore, to achieve the following two objectives. The first aim of this study was to systematically characterise the patterns of Hsp27 and −70 expression in the retina and optic nerve in four discrete models of retinal ganglion cell (RGC) degeneration: axonal injury (ON crush), somato-dendritic injury (NMDA-induced excitotoxicity), chronic hypoperfusion (bilateral occlusion of the carotid arteris) and experimental glaucoma. The second aim was to document Hsp27 and −70 expression in the optic tract, the subcortical retinorecipient areas of the brain, and the visual cortex during Wallerian degeneration of RGC axons. Hsp27 was robustly upregulated in the retina in each injury paradigm, with the chronic models, 2VO and experimental glaucoma, displaying a more persistent Hsp27 transcriptional response than the acute models. Hsp27 expression was always associated with astrocytes and with a subset of RGCs in each of the models excluding NMDA. Hsp27 was present within astrocytes of the optic nerve/optic tract in control rats. During Wallerian degeneration, Hsp27 was upregulated in the optic nerve/optic tract and expressed *de novo* by astrocytes in the lateral geniculate nucleus and the stratum opticum of the superior colliculus. Conversely, the results of our study indicate Hsp70 was minimally induced in any of the models of injury, either in the retina, or in the optic nerve/optic tract, or in the subcortical, retinorecipient areas of the brain. The findings of the present study augment our understanding of the involvement of Hsp27 and Hsp70 in the response of the visual system to RGC degeneration.

## Introduction

One mechanism by which mammalian cells respond to pathophysiological stress is via the synthesis of highly conserved molecular chaperones termed heat shock proteins (Hsps). These inducible proteins confer cellular protection against ongoing and subsequent insults, as well as contributing to cellular repair mechanisms [1]. Hsp27 and Hsp70 are among the most highly inducible members of the Hsp family and, without doubt, the most studied, not least because of their potent anti-apoptotic properties. In the brain, induction of both Hsp27 and −70 occurs within defined spatial and temporal parameters in response to various pathological conditions, including, but by no means restricted, to ischemia, excitotoxicity and axonal injury [2]. These Hsps are increasingly viewed as ideal candidates to serve as biomarkers to identify near-lethal stress associated with pathological conditions in the brain [3]. In terms of the cell types involved in each response, Hsp27 is principally upregulated by astrocytes, while Hsp70 is classically expressed by neurons [2].

Loss of retinal ganglion cells (RGCs) and degeneration of their axons occurs in a variety of conditions, most notably glaucomatous optic neuropathy, but also anterior ischemic optic neuropathy, arterial and venous vessel occlusions, traumatic optic neuropathy and diabetic retinopathy. There is a strong likelihood that Hsp27 and/or Hsp70 are upregulated as endogenous survival factors in conditions involving RGC loss, as occurs in neurodegenerative paradigms in the brain. It is surprising, therefore, to find that no coherent picture has emerged regarding the magnitude, duration and cellular distribution of Hsp70 in models of RGC degeneration. While some authors have documented an upregulation of Hsp70 by RGCs in paradigms of ischemia [4], [5], [6], other studies have found no evidence for increased expression [7], [8]. In an analagous way, studies utilising induced rat models of experimental glaucoma, have produced opposing results concerning whether or not Hsp70 is induced [9], [10]. Furthermore, an increase in Hsp70 expression by RGCs has been noted after excitotoxic injury [11], but not in a model of ischemic optic neuropathy [12]. The situation as regards Hsp27 is less ambiguous. In contrast to the brain, Hsp27 appears to be upregulated both by neurons and glial cells in models of RGC degeneration [7], [13], [14], [15], [16].

There is increasing recognition that optic neuropathies, such as glaucoma, affect the visual target areas in the brain at early stages of the disease. While transsynaptic degeneration of neurons within the LGN and SC following Wallerian degeneration of RGC axons appears to be a protracted process, glial cell activation [17], [18], axonal transport deficits [19], [20], [21] and dendritic changes [22] have all been shown to be early events. One recent study found that positron emission tomography imaging of an activated glial marker in the LGN of glaucomatous monkeys was a sensitive and early tool for disease diagnosis [23]. An increased understanding of the pathology of the visual target areas during RGC axonal inury will potentially aid disease diagnosis, provide tissue biomarkers to more precisely assess injury status, and better inform as to suitable neuroprotection strategies. In principal, inducible Hsps represent ideal candidates for each of these goals; however, to date, little information has been available à propos the expression patterns of inducible Hsps in the visual target areas of the brain during Wallerian degeneration of RGC axons.

The current study sought to achieve two objectives. The first aim was to systematically characterise the patterns of Hsp27 and −70 expression in the retina and ON in four distinct rat models of RGC degeneration: (1) Optic nerve (ON) crush, which causes severe mechanical trauma to myelinated RGC axons and leads to retrograde death of RGCs; (2) NMDA-induced excitotoxicity, which induces somato-dendritic death of RGCs; (3) Bilateral occlusion of the common carotid arteries (2VO), which results in chronic retinal and ON hypoperfusion; (4) Laser-induced experimental glaucoma. The second aim was to document Hsp27 and −70 expression in the optic tract (OT), the subcortical retinorecipient areas of the brain, and the visual cortex during Wallerian degeneration of RGC axons. To achieve these goals, we used a combination of qPCR, immunohistochemistry and Western blotting.

## Materials and Methods

### Animals and procedures

This study was approved by the South Australia Pathology/Central Health Network Animal Ethics committee (permit numbers: 117/11, 97/08) and conforms with the Australian Code of Practice for the Care and Use of Animals for Scientific Purposes, 2004. All procedures were performed under anaesthesia (100 mg/kg ketamine and 10 mg/kg xylazine), and all efforts were made to minimize suffering. All experiments also conformed to the ARVO Statement for the Use of Animals in Ophthalmic and Vision Research. Adult Sprague-Dawley rats (220–300 g) were housed in a temperature- and humidity-controlled environment with a 12-hour light, 12-hour dark cycle and were provided with food and water ad libitum.

#### Experimental glaucoma

Ocular hypertension was induced in the right eye of each animal by laser photocoagulation of the trabecular meshwork using a slightly modified protocol [24] of the method described by Levkovitch-Verbin et al. [25]. For the two week time point, a second laser treatment was given on day 7 if the difference in intraocular pressure (IOP) between the two eyes was less than 8 mmHg. IOPs were measured in both eyes at baseline, day 1, day 3, day 7 and day 14 using a rebound tonometer, factory calibrated for use in rats. All animals demonstrated an adequate IOP elevation (minimum increase in IOP of 10 mmHg). Two animals were excluded as a result of death under anaesthesia and 2 due to hyphema. The number of rats analysed at each time point for RT-PCR was as follows: 1d (*n* = 7), 3d (*n* = 8), 7d (*n* = 8), 14d (*n* = 7). The retinas and ONs from the 7d cohort were also used for Western blotting. In addition, a further 3 animals were taken at each time point for immunohistochemistry.

#### NMDA-induced excitotoxicity

For NMDA experiments, an intravitreal injection of 40 nmol of NMDA (5 µl in sterile saline) was performed in one eye, while the contralateral eye received 5 µl of sterile saline. The number of rats analysed at each time point for RT-PCR was as follows: 1d (*n* = 6), 3d (*n* = 7), 7d (*n* = 7). The retinas and ONs from the 7d cohort were also used for Western blotting. In addition, a further 3 animals were taken at each time point for immunohistochemistry.

#### ON crush

ON crush was performed as previously described [26]. In brief, the superior muscle complex was divided and the ON exposed by blunt dissection. The ON was then crushed 3 mm posterior to the globe under direct visualisation using number 5 forceps for 20s. To avoid confusing retinal ischemic changes with the effects of crush, the fundus was observed ophthalmoscopically immediately after nerve crush, in order to discard any animals with pale (ischemic) retina as a result of surgery. The number of rats analysed at each time point for RT-PCR was as follows: 1d (*n* = 4), 3d (*n* = 4), 7d (*n* = 4). The retinas and ONs from the 7d cohort were also used for Western blotting. In addition, a further 3 animals were taken at each time point for immunohistochemistry.

#### 2VO

Bilateral occlusion of the common carotid arteries was performed as previously described [27]. In brief, rats were anaesthetised and a ventral incision was made. The common carotid arteries were then bilaterally separated from the carotid sheath and vagus nerve. The arteries were ligated with silk sutures. Sham animals received the same operation without occlusion of the vessels. The number of rats analysed at each time point for RT-PCR was as follows: sham (*n* = 8), 2d (*n* = 8), 7d (*n* = 8). The retinas and ONs from the 7d cohort were also used for Western blotting. In addition, a further 3 animals were taken at each time point for immunohistochemistry.

### Tissue Processing and Immunohistochemistry

All rats were killed by transcardial perfusion with physiological saline under deep anaesthesia and, in those rats where tissue was not taken for RT-PCR/Western blotting, subsequently with 10% buffered formalin. The brain, both eyes, both ONs and the optic chiasm were carefully dissected. For rats taken for immunohistochemsitry, all tissues were then fixed in 10% buffered formalin for at least 24 h. Following fixation, the brain was positioned in the Kopf Rat brain blocker (Kopf Instruments PA001) and 2 mm coronal slices were taken starting from the dorsal and proceeding to the caudal portion of the brain. Brain slices, along with the globe and optic pathway, were then processed for routine paraffin-embedded sections [28]. Globes were embedded sagitally, while ONs and chiasmata were embedded longitudinally. In all cases, 4 µm serial sections were cut using a rotary microtome.

Colourimetric immunohistochemistry was performed as previously described [26], [28]. Briefly, tissue sections were deparaffinised and endogenous peroxidase activity was blocked with H_2_O_2_. Antigen retrieval was performed by microwaving sections in 10 mM citrate (pH 6.0) and non-specific labelling blocked with PBS containing 3% normal horse serum (PBS-NHS). Sections were incubated overnight at room temperature in primary antibody (in PBS-NHS, see Table 1), followed by consecutive incubations with biotinylated secondary antibody (Vector, Burlingame, CA) and streptavidin-peroxidase conjugate (Pierce, Rockford, IL). Color development was achieved using either NovaRed substrate kit (Vector, Burlingame, CA) for 3 min or 3′-,3′-diaminobenzidine for 5 min. Sections were counterstained with hematoxylin, dehydrated, cleared in histolene and mounted. Confirmation of the specificity of antibody labelling was judged by the morphology and distribution of the labelled cells, by the absence of signal when the primary antibody was replaced by isotype/serum controls, by comparison with the expected staining pattern based on our own, and other, previously published results, and, by the detection within retinal samples of a protein at the expected molecular weight by Western blotting.

**Table 1:** Antibodies.

Protein	Source	Clone/Cat. No.	Species	Immunogen	Dilution
Actin	Sigma	clone AC-15	Mouse	Slightly modified β-cytoplasmic actin N-terminal peptide	1∶20,000^W^
Calbindin	Sigma	Clone CB-955	Mouse	purified bovine kidney calbindin-D-28K	1∶500
GFAP	Dako	Clone 6F2	Mouse	GFAP from human brain	1∶200^f^, 1∶1000
Glutamine synthetase	BD Transduction	Cat# 610517	Mouse	Sheep glutamine synthetase aa. 1–373	1∶1000^f^
Hsp27	Enzo Life Sciences	Cat# ADI-SPA-801	Rabbit	recombinant mouse Hsp25 protein	1∶2500, 1∶400^f^, 1∶1000^W^
Hsp70	Enzo Life Sciences	Cat# ADI-SPA-810	Mouse	native human Hsp70 protein	1∶400, 1∶1000^W^
Iba1	Wako	019-19741	Rabbit	Synthetic peptide corresponding to the Iba1carboxy-terminal sequence	1∶4000^f^, 1∶20,000
NeuN	Millipore	clone A60	Mouse	Purified cell nuclei from mouse brain	1∶1000
Parvalbumin	Sigma	Clone PARV-19	Mouse	Purified frog muscle parvalbumin	1∶2000
PCNA	Dako	clone PC10	Mouse	Rat PCNA-protein A fusion protein obtained from vector pC2T	1∶2000^f^
Np-NFH	Covance	Clone SMI-32	Mouse	Homogenized hypothalmi recovered from Fischer 344 rats	1∶10,000
χ-synuclein	^†^DSHB	Cat# CPTC-SNCG-1 (supernatant)	Mouse	recombinant full-length human χ-synuclein	1∶40^f^
vimentin	Dako	clone V9	Mouse	Purified vimentin from porcine eye lens	1∶200^f^

f dilution used for 2-step fluorescent immunostaining procedure;

W dilution used for Western blotting;^ †^Developmental Studies Hybridoma Bank.

For double labelling fluorescent immunohistochemistry, visualisation of one antigen was achieved using a 3-step procedure (primary antibody, biotinylated secondary antibody, streptavidin-conjugated AlexaFluor 488 or 594), while the second antigen was labelled by a 2-step procedure (primary antibody, secondary antibody conjugated to AlexaFluor 488 or 594). Sections were prepared as above, then incubated overnight at room temperature in the appropriate combination of primary antibodies. On the following day, sections were incubated with the appropriate biotinylated secondary antibody for the 3-step procedure plus the correct secondary antibody conjugated to AlexaFluor 488 or 594 for the 2-step procedure, followed by streptavidin-conjugated AlexaFluor 488 or 594. Sections were then mounted using anti-fade mounting medium and examined under a confocal fluorescence microscope.

### Evaluation of immunohistochemistry in the LGN and SC

Quantitative evaluation of Hsp27 and Hsp70 expression in the LGN and SC was conducted in animals previously subjected to ON crush and in control rats.

#### LGN

Initially, coronal sections were stained for H&E and immunolabelled for parvalbumin in order to verify that the correct anatomical position had been identified. Parvalbumin facilitates easy identification of the dorsal LGN (dLGN), ventral LGN (vLGN) and intergeniculate leaflet (IGL). The brain area of interest was set at approximately bregma −4.5 mm, the midpoint of the LGN [29]. Subsequently, sections were labelled for Hsp27 and Hsp70. Next, the number of Hsp27- and Hsp70-positive cells were counted manually by a single observer in photomicrographs taken in the dLGN and vLGN regions of both the ipsilateral and contralateral sides. Selection criteria excluded small cell fragments or cells without a visible nucleus. The area photographed and used for quantification (350 µm×260 µm) represents 0.091 mm^2^ and was set around the central portion of the dLGN and the lateral division of the vLGN. Three stained sections were quantified from each animal.

#### SC

Initially, coronal sections were stained for H&E and immunolabelled for calbindin and parvalbumin in order to verify that the correct anatomical position had been identified. The brain area of interest was set at approximately bregma −6.0 mm [29]. Sections were then labelled for Hsp27 and Hsp70 and the number of positive cells were counted in photomicrographs taken in the stratum opticum/stratum griseum superficiale of the ipsilateral and contralateral SC. From each immunolabelled section, 3 fields were selected, representing the medial through to lateral SC. As for the LGN, 3 stained sections were typically quantified from each animal. The area photographed and used for quantification (350 µm×260 µm) in each case equates to 0.091 mm^2^.

For both the LGN and SC, all measurements from one animal were averaged and treated as an independent data point. All data are presented as mean ± SEM and statistical comparisons were made between the control, 3d and 7d groups by ANOVA, followed by post-hoc Dunnett's Multiple Comparison Test.

### Western Immunoblotting

Electrophoresis/Western blotting was performed as previously described [27]. In brief, tissue extracts were sonicated in homogenisation buffer, diluted with an equal volume of sample buffer, and boiled for 3 minutes. Electrophoresis was performed using non-denaturing 12% polyacrylamide gels. After separation, proteins were transferred to polyvinylidine fluoride membranes for immunoprobing. Blocking of membranes was carried out in a solution of tris-buffered saline (TBS) containing 0.1% (v/v) Tween-20 and 5% (w/v) non-fat dried skimmed milk (TBS-TM). Membranes were then incubated consecutively with the appropriate primary antibody (Table 1), biotinylated secondary antibody and streptavidin-peroxidase conjugate. Colour development was achieved using 3-amino-9-ethylcarbazole. The images were captured and analysed for densitometry using the program, Adobe PhotoShop CS2. Densitometry values were normalised for actin. Statistical analysis was carried out by Student's unpaired t-test (control group vs 7d injured group). The null hypothesis tested was that densitometry measurements for target proteins (normalised for actin) would be the same in control and experimental retinas.

### Real-time RT-PCR

Real time PCR studies were carried out essentially as described previously [30]. In brief, tissues were dissected, total RNA was isolated and first strand cDNA was synthesised from DNase-treated RNA samples. Real-time PCR reactions were carried out in 96-well optical reaction plates using the cDNA equivalent of 20 ng total RNA for each sample in a total volume of 20 µl containing 1× SYBR Green PCR master mix (BioRad), forward and reverse primers. Thermal cycling conditions were 95°C for 3 min and 40 cycles of amplification comprising 95°C for 12 s, appropriate annealing temperature for 30 s and 72°C for 30 s. Primer sets used were as follows (sense primer, antisense primer, annealing temperature, product size, accession number): GAPDH (5′-TGCACCACCAACTGCTTAGC-3′, 5′-GGCATGGACTGTGGTCATGAG-3′, 63°c, 87 bp, GenBank ID: NM_017008), NF-L (5′-ATGGCATTGGACATTGAGATT-3′, 5′-CTGAGAGTAGCCGCTGGTTAT-3′, 63°c, 105 bp, AF031880), Hsp27 (5′- CACTGGCAAGCACGAAGAAA-3′, 5′-CAGGGGACAGGGAAGAGGA-3′, 62°c, 119 bp, GenBank ID: NM_031970), Hsp70 (5′-ACGAGGGTCTCAAGGGCAAG-3′, 5′-CTCTTTCTCAGCCAGCGTGTTAG-3′, 63°c, 107 bp, GenBank ID: NM_031971). PCR assays were performed using the IQ5 icycler (Bio-Rad) and all samples were run in duplicate. GAPDH, NF-L, Hsp27 and Hsp70 mRNAs amplified with high efficiency and linearity during real-time PCR. Mean amplification efficiencies, as determined by plotting cycle threshold as a function of initial cDNA quantity, were approximately 2.00 for GAPDH, NF-L, Hsp27 and Hsp70. Results obtained from the real-time PCR experiments were, therefore, quantified using the comparative threshold cycle (C_T_) method (ΔΔC_T_) for relative quantitation of gene expression [31]. All values were normalised using the endogenous housekeeping gene GAPDH and expressed relative to controls. Statistical analysis was carried out by one-way ANOVA followed by post-hoc Dunnett's Multiple Comparison Test (NMDA, ON crush, 2VO) or by Student's unpaired t-test followed by modified Bonferroni correction (experimental glaucoma). The null hypothesis tested was that C_T_ differences between target and housekeeping genes would be the same in control and experimental retinas.

## Results

### Expression of Hsp27 and Hsp70 in brain after transient MCAO

Tissue sections from rats subjected to focal ischemia were employed in this study for two reasons: Firstly, transient MCAO is well-known to cause a pronounced induction of both Hsp27 and Hsp70 in the affected hemisphere [32], [33]. As such, the model serves as an excellent positive control for the specificity of the Hsp27 and −70 antibodies. Secondly, the results provide a useful tool by which to compare the cell types responsible for expression of Hsp27 and −70 in the brain and retina after neuronal injury.

**Figure 1:**
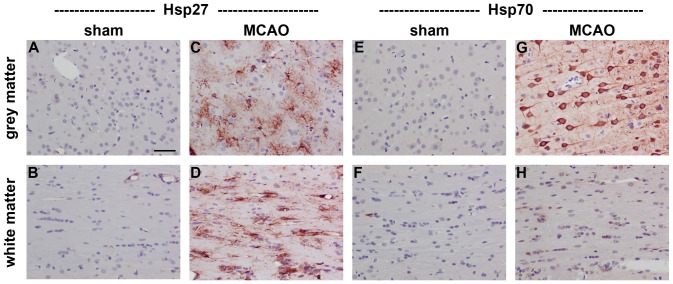
Representative images of Hsp27 and Hsp70 expression in rat brain after transient MCAO. Hsp27 immunolabelling is restricted to blood vessels in sham brains (A, B), but is widely expressed by reactive astrocytes in ipsilateral grey and white matter regions at 1d following MCAO (C, D). Hsp70 immunolabelling is minimal in sham brains (E, F), but is exclusively associated with neurons in ipsilateral grey matter regions at 1d following MCAO (G). No unambiguous Hsp70 labelling is evident in white matter tracts (H). Note: grey matter, neocortex; white matter, corpus callosum; MCAO, middle cerebral artery occlusion. Scale bar  = 50 µm.

In sham brains, Hsp27 immunolabelling was restricted to some blood vessels and to very occasional astrocytes in white matter tracts, while there was minimal constitutive expression of Hsp70 in either grey or white matter (Fig. 1). Transient MCAO, which causes an ischemic infarct centred within the striatum, resulted in a dramatic induction of both Hsp27 and Hsp70 at 1d in the affected hemisphere. Expression of Hsp27 encompassed both grey and white matter and was overwhelmingly associated with cells with the morphology of astrocytes (Fig. 1). In contrast, Hsp70 was expressed by cells with the morphology of neurons, but was ostensibly absent from white matter tracts. Double labelling immunofluorescence confirmed that Hsp27 expression colocalised with GFAP-labelled astrocytes, while Hsp70 colocalised to NeuN-labelled neurons (data not shown).

### Expression of Hsp27 and Hsp70 mRNAs in the retina during RGC degeneration

**Figure 2:**
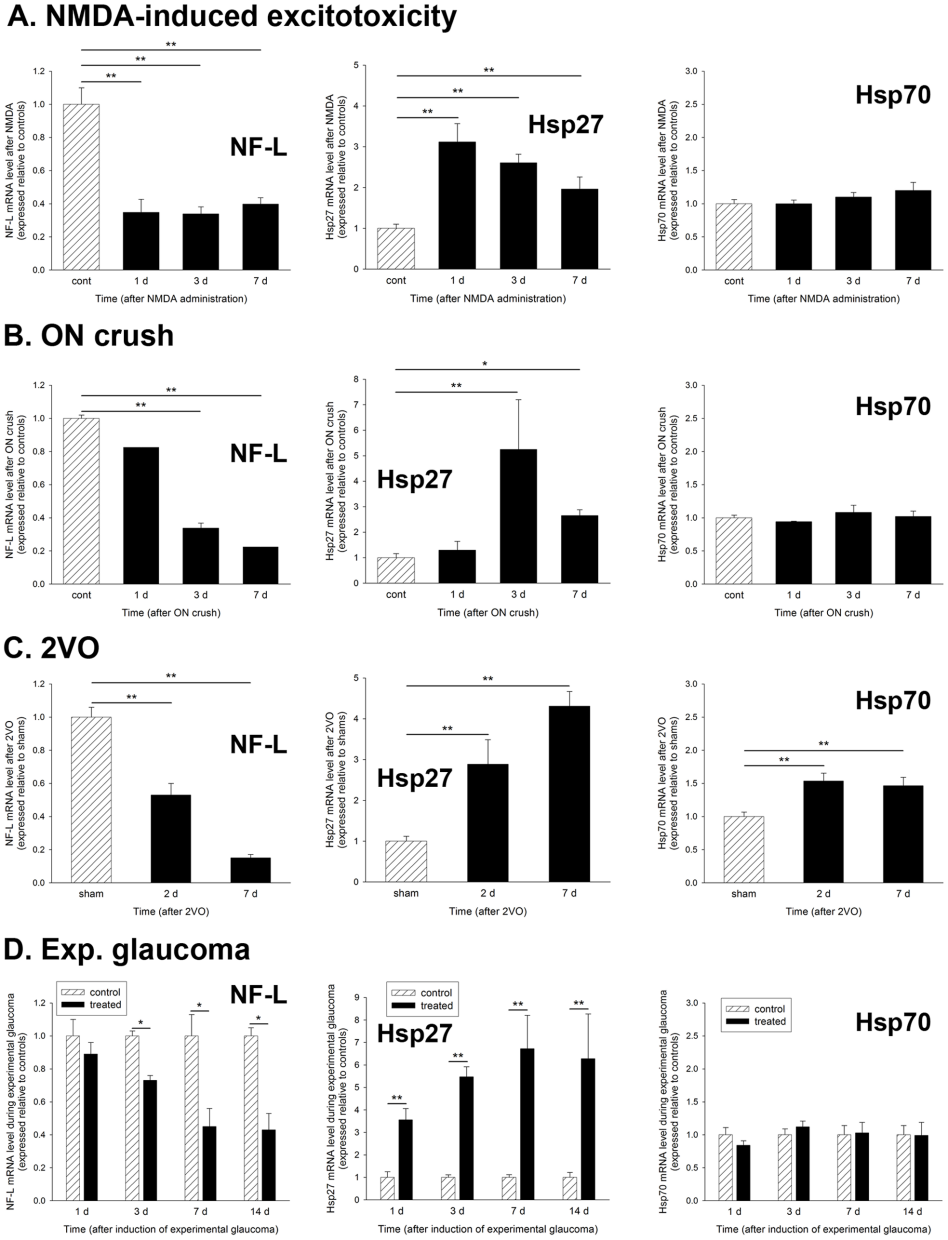
Temporal characterization of NF-L, Hsp27 and Hsp70 mRNAs in the retina during RGC injury. Values (represented as mean ± SEM) are normalised for GAPDH and expressed relative to the control group. *P<0.05, **P<0.01 by one-way ANOVA followed by post-hoc Dunnett's Multiple Comparison Test (NMDA, ON crush, 2-VO) or by Student's unpaired t-test followed by modified Bonferroni correction (experimental glaucoma). NF-L, neurofilament light; 2VO, bilateral occlusion of the common carotid arteries.

We performed a temporal characterization of Hsp27 and Hsp70 gene expression in the retina in four models of RGC degeneration. To impart perspective on the results, we also determined the levels of neurofilament light (NF-L) mRNA, which is a sensitive early gauge of RGC viability [34], [35]. After NMDA-induced excitotoxicity (Fig. 2A), NF-L mRNA was maximally down-regulated by 1d, indicating that all affected RGC somas were no longer transcriptionally viable. Hsp27 mRNA was upregulated 3.1-fold at this time point, then slowly declined towards the control level at the 3d and 7d time points. Hsp70 mRNA was not statistically different from controls at any time point. Following ON crush (Fig. 2B), NF-L mRNA was statistically unaffected at 1d, but was reduced to 34% and 23% of the control level at 3d and 7d, respectively. Hsp27 mRNA was not changed at the earliest time point, but was 5.2-fold higher than controls at 3d and 2.6 fold higher at 7d. Hsp70 mRNA was not statistically different from controls at any time point after ON crush. During 2VO (Fig. 2C), the NFL mRNA level in treated retinas was 53% of the level in shams at 2d, and 15% by 7d. Hsp27 mRNA was 2.9-fold higher than shams at 2d, and 4.3 fold higher at 7d. In contrast to the other models of injury, there was a small (1.5-fold at both 2d and 7d), but statistically significant (P<0.01 for both time points), upregulation of Hsp70 mRNA during 2VO. After induction of experimental glaucoma (Fig. 2D), the NFL mRNA level in treated retinas was 89% of controls at 1d, 73% at 3d, 45% at 7d, and 43% by 14d. As regards Hsp27, there was a striking and sustained increase in transcription, such that the Hsp27 mRNA level was 3.5-fold higher than controls at 1d, 5.5-fold at 3d, 6.7-fold at 7d, and 6.3-fold at 14d. Again, Hsp70 mRNA was unaffected at any time point during experimental glaucoma.

### Expression of Hsp27 and Hsp70 proteins in the retina during RGC degeneration

**Figure 3:**
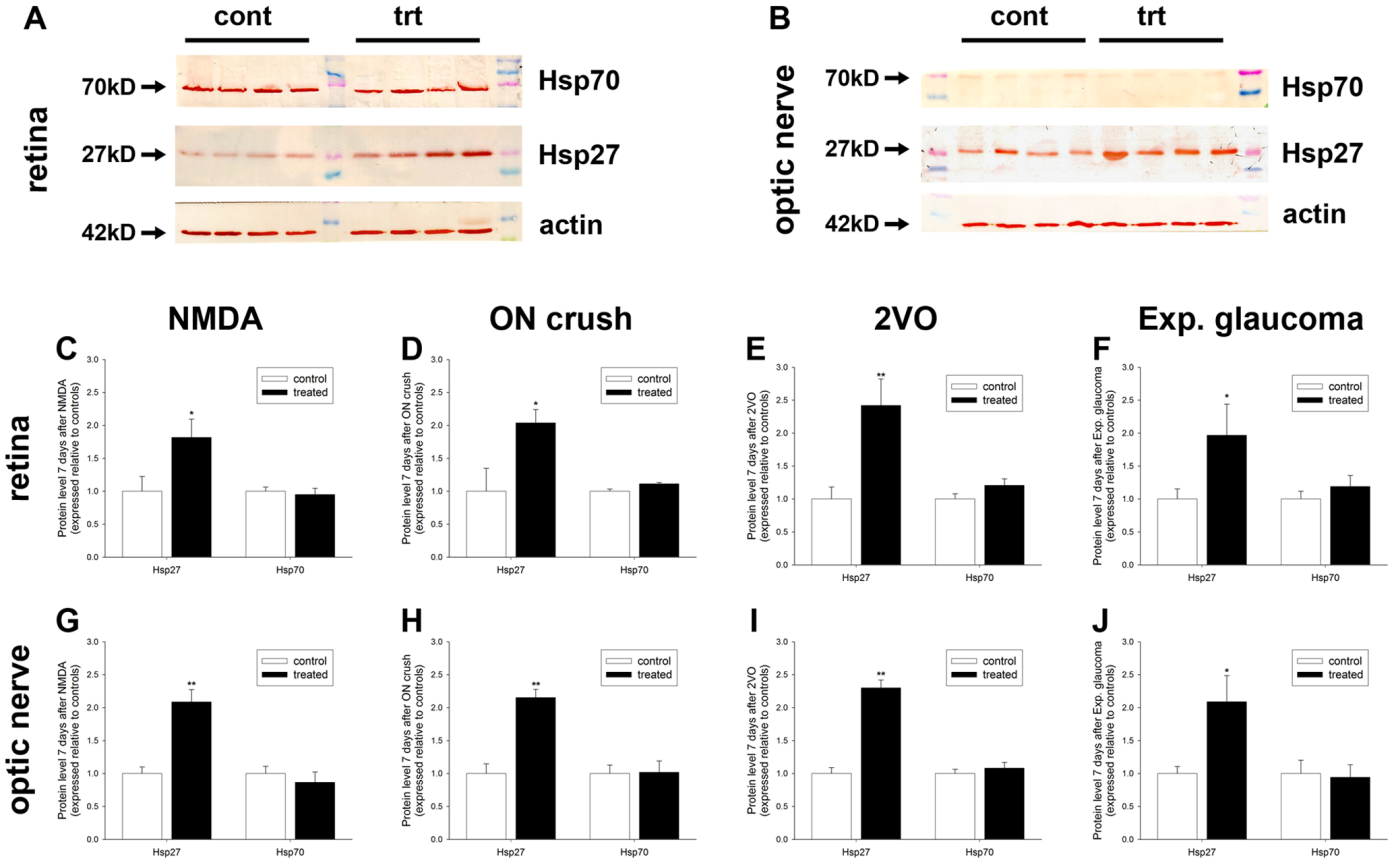
Characterization of Hsp27 and Hsp70 proteins in the retina and optic nerve during RGC injury. A, B: Representative Hsp27, Hsp70 and actin immunoblots in control (cont) and injured (trt) samples. Single bands of the expected molecular weight are apparent. C-J: Levels of Hsp27 and Hsp70 proteins. Values (represented as mean ± SEM) are normalised for actin and expressed relative to controls. *P<0.05, **P<0.01 by Student's unpaired t-test.

When incubated with retinal samples from control or treated rats, the Hsp27 and Hsp70 antibodies each detected a single band at the predicted molecular weight (27 kD and 70 kD, respectively) by Western blotting (Fig. 3A). Inspection of the blots indicated that the level of Hsp70 protein in healthy retinas is, in all probability, higher than the level of Hsp27 protein, as the former band was always more intense than the latter in any given sample. Quantification of the levels of Hsp27 and Hsp70 proteins at the 7d time point in the various models of RGC injury revealed that Hsp27 was significantly (*P*<0.01 for 2VO; *P*<0.05 for other models) upregulated in the retinas from all four treated groups compared to the relevant controls (Fig. 3C–F). The extent of Hsp27 upregulation in each model was broadly similar, with NMDA, as was the case for the mRNA study, proving slightly less efficacious than the other injury models. As regards Hsp70 protein, no significant upregulation was found in any RGC injury model, although treated retinas in the 2VO model showed a tendency (21% higher than controls; *P* = 0.18) to higher expression.

**Figure 4:**
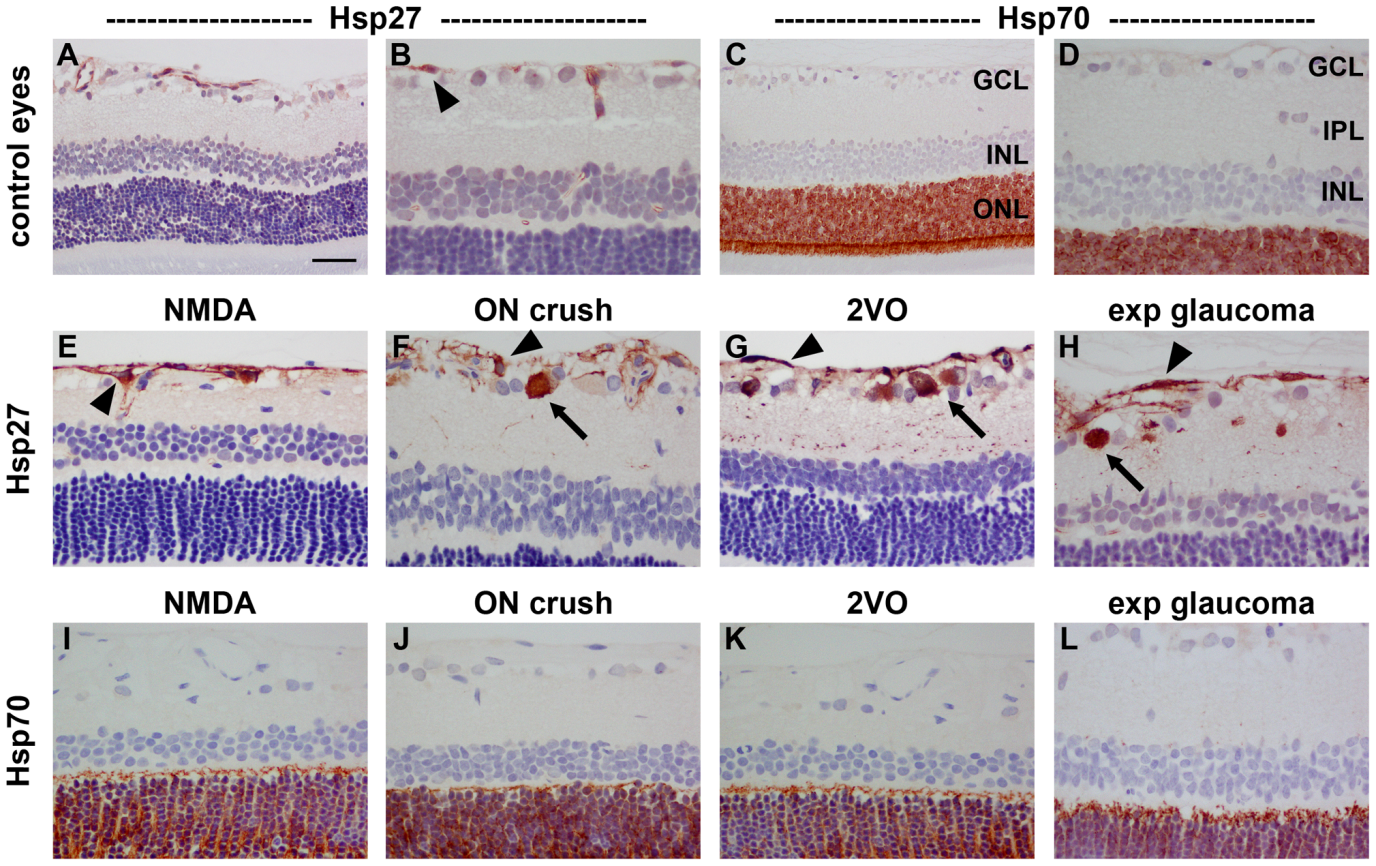
Representative images of Hsp27 and Hsp70 expression in control and 7d injured retinas. In control retinas (A, B), Hsp27 is weakly associated with blood vessels and astrocytes (arrowhead), but negligible labelling is evident the other layers of the retina. In all four models of RGC injury (E–H), Hsp27 is upregulated in astrocytes (arrowhead). Following induction of ON crush, 2VO and experimental glaucoma, but not NMDA, Hsp27 is also associated with occassional cells in the GCL and their trailing processes in the IPL. In control retinas (C, D), Hsp70 is exclusively localised to photoreceptors in the ONL (arrowhead). No alteration to the pattern of Hsp70 is apparent in any of the four models of RGC injury (I–L). GCL, ganglion cell layer; INL, inner nuclear layer; IPL, inner plexiform layer ONL, outer nuclear layer. Scale bar: A, C = 50 µm; B, D–L = 25 µm.

**Figure 5:**
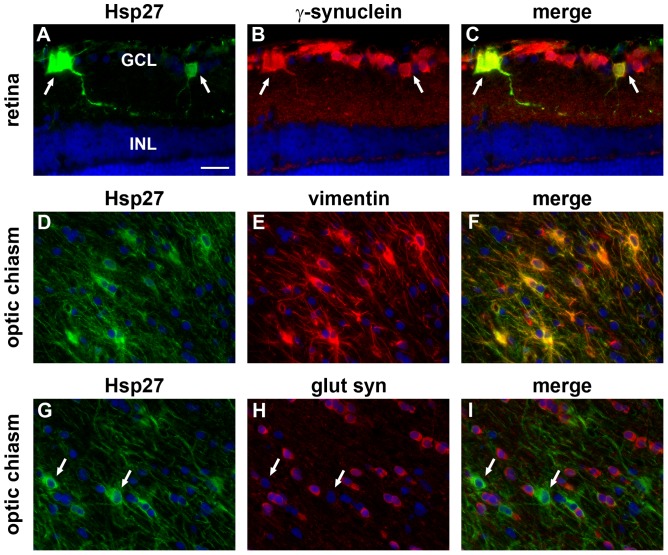
Double labelling immunofluorescence of Hsp27 in the retina and optic chiasm 7d after ON crush. In the retina, Hsp27 colocalises with the RGC marker χ-synuclein (arrows, A–C). In the optic chiasm, Hsp27 colocalises with the astrocytic marker vimentin (D–F), but not with the oligodendrocyte marker glutamine synthetase (arrows, G–I). GCL, ganglion cell layer; INL, inner nuclear layer; glut syn, glutamine synthetase. Scale bar  = 25 µm.

Immunolabelling of tissue sections from control retinas showed that Hsp27 is expressed in blood vessels and to some extent by astrocytes (Fig. 4A, B), while Hsp70 expression is localised exclusively to photoreceptor cell bodies and inner segments (Fig. 4C, D). NMDA caused a sustained increase in Hsp27 expression by astrocytes when compared with control eyes, which was detectable within 24 h (data not shown) and persisted for at least 7d (Fig. 4E). Hsp27 immunoreactivity was, however, not associated with surviving RGCs after NMDA. ON crush, 2VO and experimental glaucoma also induced persistent upregulation of Hsp27 by astrocytes (Fig. 4F–H). In addition, Hsp27-positive cells were evident in the GCL, but not the INL, from 3d onwards in each injury model (see Fig. 4F-H for representative images from 7d retinas). Double labelling immunofluorescence showed that, in each injury model, Hsp27 colocalised with χ-synuclein, indicating its presence within RGCs (See Fig. 5A–C for representative images from a rat subjected to ON crush). No discernible upregulation of Hsp70 protein was detected in RGCs, in other retinal neurons, or in retinal glial cells either at 1d (data not shown), 3d (data not shown), or 7d (Fig. 4I–L) following NMDA, ON crush, 2VO or experimental glaucoma, or at 14d after experimental glaucoma (data not shown). Constitutive expression of Hsp70 by photoreceptors appeared unaffected in all four models of injury (Fig. 4I–L).

### Expression of Hsp27 and Hsp70 in the ON and OT during RGC degeneration

When incubated with ON samples from control or treated rats, the Hsp27 antibody detected a single band at approximately 27 kD by Western blotting (Fig. 3B). The Hsp70 antibody, in contrast, elicited only a very faint band of the expected molecular weight when used with the same samples (Fig. 3B). Quantification of the levels of Hsp27 and Hsp70 proteins in the ON at the 7d time point in the various models of RGC injury revealed highly similar results to the retina, namely that Hsp27 was significantly (*P*<0.05 for experimental glaucoma; *P*<0.01 for other models) upregulated in all four treated groups compared to the relevant controls (Fig. 3G–J). The extent of Hsp27 upregulation in each model was comparable (2–2.5-fold). As regards Hsp70 protein, no significant upregulation was found in any RGC injury model (Fig. 3G–J); Hsp70 bands were faint or not detectable in all samples.

**Figure 6:**
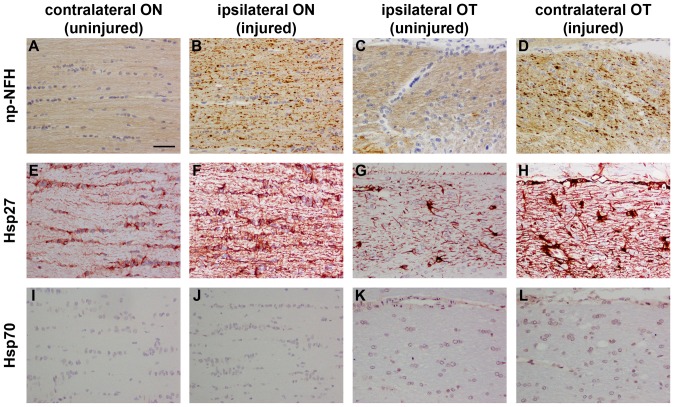
Representative images of np-NFH, Hsp27 and Hsp70 in the ON and OT after ON crush. In the uninjured pathway (contralateral ON and corresponding ipsilateral OT), a pattern of uniform labelling of RGC axons by np-NFH is largely evident (A, C). In the injured pathway (ipsilateral ON and corresponding contralateral OT), numerous swollen, beaded np-NFH-labelled axons are apparent, indicative of Wallerian degeneration (B, D). In the uninjured ON and OT, Hsp27 is associated with a population of glial cells. One week after induction of ON crush, Hsp27 expression is markedly upregulated throughout the injured pathway. No expression of Hsp70 is readily apparent in the uninjured or injured ON or OT (I–L). ON, optic nerve; OT, optic tract; Np-NFH, non-phosphorylated neurofilament heavy chain. Scale bar  = 50 µm.

Immunolabelling of tissue sections from control ONs and OTs (approximately bregma −3.3 mm) showed that Hsp27 is expressed by cells with the morphological appearance and distribution of astrocytes (Fig. 6E, G), while Hsp70 expression was not detectable (Fig. 6I, K). Analysis of ONs and OTs from rats subjected to the four models of injury showed essentially identical patterns of immunolabelling. In each case, the injury model caused Wallerian degeneration of RGC axons throughout the length of the ON and OT, which can be easily visualized at the 7d time point by labelling with an antibody that recognises non-phosphorylated neurofilament heavy chain (np-NFH). Compared with uniformly-labelled healthy axons, degenerating axons have a swollen, beaded appearance (Fig. 6A–D). In ONs and OTs from the treated groups with degenerating axons, substantially more Hsp27 immunolabelling was apparent (Fig. 6F, H). This was the case for the entire length of the white matter tract from the optic nerve head via the optic chiasm to the brachium of the superior colliculus. In contrast, no discernible immunolabelling for Hsp70 was evident in any injured ONs or OTs (Fig. 6J, L). Double labelling immunofluorescence showed that, in control tissue and in the injury models, Hsp27 in the ON and OT colocalised with astrocytic markers (See Fig. 5D–F for representative images from the optic chiasm of a rat subjected to ON crush and double labelled for Hsp27 and vimentin). No colocalisation was observed with markers specific to oligodendrocytes, such as glutamine synthetase (Fig. 5G–I, see S1 Fig. for confirmation of specificity of glutamine synthetase to oligodendrocytes).

### Expression of Hsp27 and Hsp70 in the LGN and SC during RGC degeneration

Detailed analysis of the expression of Hsp27 and −70 in the subcortical, retinorecipient areas of the brain during Wallerian degeneration of RGC axons was conducted using animals from the ON crush model of RGC injury. It should be noted, however, that a single, representative brain from each of the three other RGC injury models (NMDA, 7d; 2VO, 7d; experimental glaucoma, 14d) was analysed qualitatively and all displayed similar Hsp27 and −70 responses to those subjected to ON crush.

#### LGN

**Figure 7:**
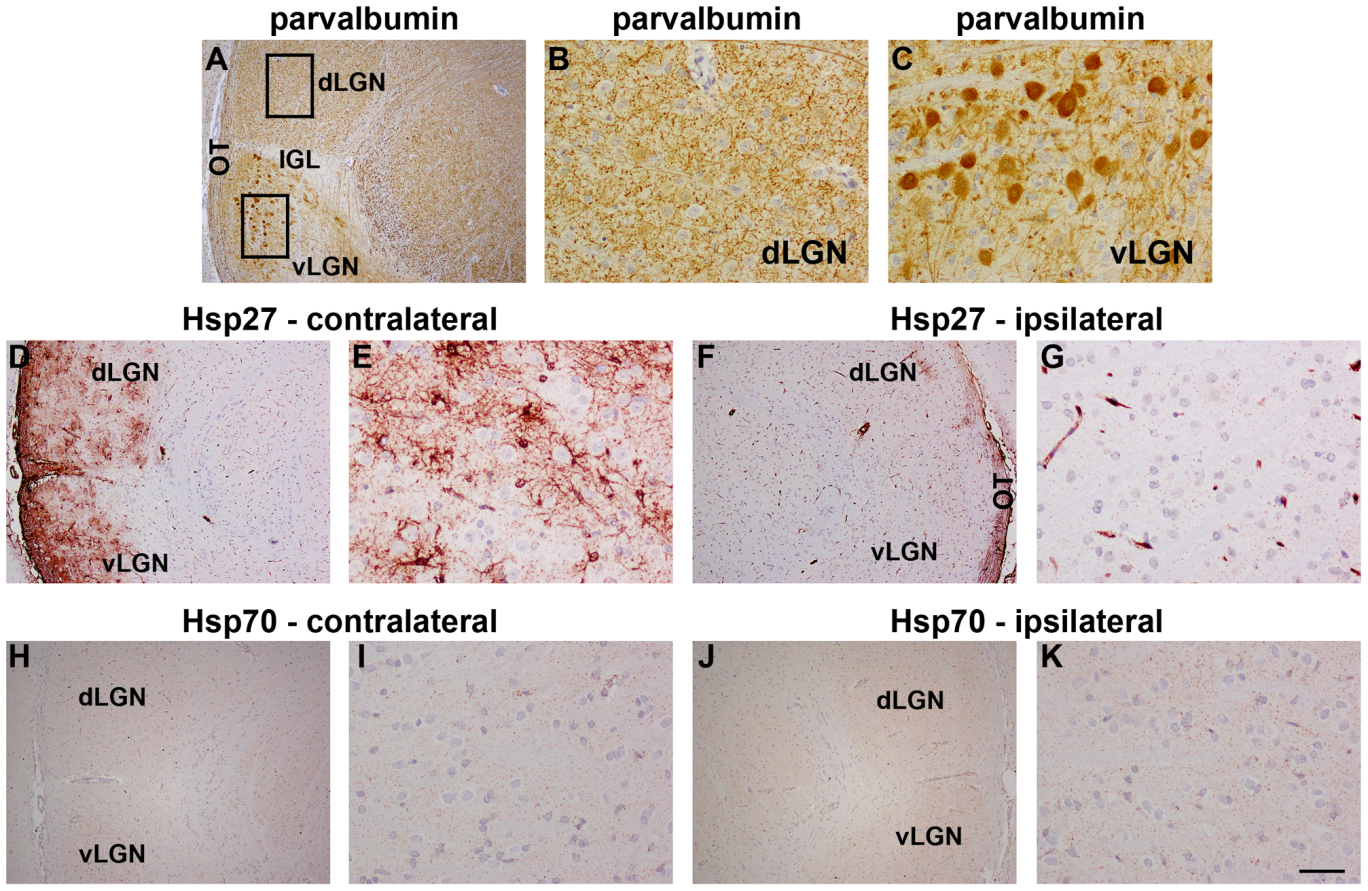
Representative images of parvalbumin, Hsp27 and Hsp70 expression in the LGN 7d after ON crush. A–C: Parvalbumin labelling in the LGN. In the dorsal LGN (dLGN), parvalbumin immunoreactivity is fine and punctate (A, B). In the ventral LGN (vLGN), numerous labelled perikarya are present (A, C). Parvalbumin immunoreactivity is absent from the intergeniculate leaflet (IGL, A). D–G: Hsp27 labelling in the LGN. Hsp27 immunoreactivity is strikingly upregulated in the injured (contralateral) LGN (D, E) when compared to the uninjured (ipsilateral) side (F, G). H–K: Hsp70 labelling in the LGN. Hsp70 immunoreactivity is uniformly low throughout the injured (contralateral; H, I) and uninjured (ipsilateral; J, K) LGN. LGN, lateral geniculate nucleus. Scale bar: A, D, F, H, J = 250 µm; B, C, E, G, I, K = 50 µm.

Immunolabelling of control animals, and animals subjected to ON crush 1d previously, showed Hsp27 expression to be restricted to blood vessels (data not shown). In 3d and 7d ON crush rats, however, the contralateral LGN additionally featured numerous Hsp27-positive cells with the apparent morphology of astrocytes distributed throughout the dLGN, IGL and vLGN (Fig. 7D, E). The ipsilateral LGN was essentially devoid of Hsp27-immunopositive cells (Fig. 7F, G). This is unsurprising as 90–95% of ON axons in the rat cross the midline and project to the contralateral OT. Quantification of the Hsp27 response in the dLGN and lateral division of the vLGN (Table 2) showed that both areas featured a similar number of labelled cells at each of the time points. Pooled (vLGN and dLGN combined) analysis of the 3d versus 7d time points did, however, show a slight increase in the number of Hsp27-positive cells at the later time (*P*<0.01 by Student's unpaired t-test). In contrast to Hsp27, no immunolabelling for Hsp70 was detectable in the dLGN, vLGN or IGL of control rats (data not shown) or ON crush rats (Fig. 7H–K). Double labelling immunofluorescence showed that Hsp27 colocalised with GFAP in the LGN (S2A–C Fig.), confirming its presence within astrocytes. No colocalisation was observed with iba1, which is specific to microglia (S2D–F Fig.).

**Table 2:** Number of of Hsp27-positive cells in the lateral geniculate nucleus after ON crush.

Time	dLGN		vLGN	
	ipsilateral	contralateral	ipsilateral	contralateral
control	0.5±0.0	0.0±0.0	0.8±0.2	0.3±0.2
3 days	0.2±0.2	21.5±2.1†	0.4±0.4	15.8±3.5†
7 days	0.8±0.3	25.7±1.8†	0.8±0.3	28.0±1.6†

Data are expressed as mean ± SEM and represent an area of 0.091 mm^2^. dLGN, dorsal lateral geniculate nucleus; vLGN, ventral lateral geniculate nucleus.

† *P*<0.01 by one-way ANOVA followed by post-hoc Dunnett's Multiple Comparison Test (control vs 3d, control vs 7d).

#### SC

**Figure 8:**
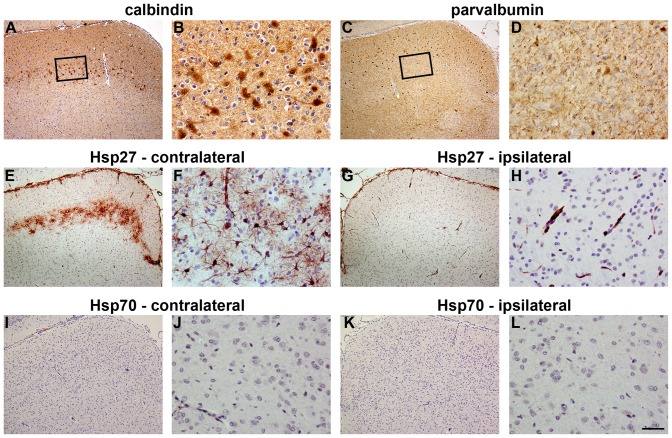
Representative images of calbindin, parvalbumin, Hsp27 and Hsp70 in the SC 7d after ON crush. A–D: Calbindin and parvalbumin labelling in the SC. The characteristic trait of calbindin immunolabelling in the SC is a band of large cell bodies that are principally localised to the stratum opticum (A, B). The distinguishing feature of parvalbumin immunolabelling in the SC is a population of smaller sized cells that are chiefly localised to the stratum griseum superficiale. The stratum opticum contains parvalbumin-positive fibres, but only scattered cells (C, D). E–H: Hsp27 labelling in the SC. Hsp27 immunoreactivity is manifest in a tight band stretching across the injured (contralateral) SC (E, F), but is negligible in the uninjured (ipsilateral) SC (G, H). I–L: Hsp70 labelling in the SC. Hsp70 immunoreactivity is uniformly low throughout the injured (contralateral; I, J) and uninjured (ipsilateral; K, L) SC. SC, superior colliculus. Scale bar: A, C, E, G, I, K = 250 µm; B, D, F, H, J, L = 50 µm.

As expected, immunolabelling of control rats, or rats subjected to ON crush 1d previously, showed Hsp27 expression to be limited to blood vessels (data not shown). In 3d and 7d ON crush rats, however, numerous Hsp27-positive cells with the morphological apperance of astrocytes were observed in a tight band localised within the superficial layers of the contralateral SC. These cells spanned the medial through to the lateral SC (Fig. 8E, F) and were evident throughout the rostral-caudal axis. As expected, very few Hsp27-labelled cells were evident in the ipsilateral SC (Fig. 8G, H). Quantification of the Hsp27 response in the SC (Table 3) revealed a very similar number of Hsp27-positive cells at 3d and 7d. As was the case for the LGN, no immunolabelling for Hsp70 was detectable in the SC of control rats (data not shown) or ON crush rats (Fig. 8I–L).

**Table 3:** Number of of Hsp27-positive cells in the superior colliculus after ON crush.

Time	superior colliculus	
	ipsilateral	contralateral
control	0.0±0.0	0.1±0.1
3 days	0.8±0.3	25.1±1.5†
7 days	1.0±0.2	26.0±1.8†

Data are expressed as mean ± SEM and represent an area of 0.091 mm^2^.

† *P*<0.01 by one-way ANOVA followed by post-hoc Dunnett's Multiple Comparison Test (control vs 3d, control vs 7d).

**Figure 9:**
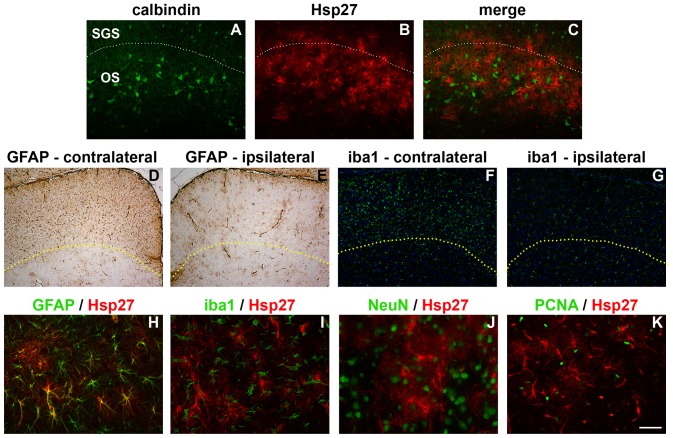
Spatial distribution and cellular localisation of Hsp27 in the SC 7d after ON crush. In the contralateral (injured) SC, the majority of Hsp27 immunolabelling is localised to the stratum opticum, as delineated by comparison to calbindin (A–C), where the dashed line indicates the accepted boundary between the stratum griseum superficiale and stratum opticum. In contrast, both GFAP (D, E) and iba1 (F, G) are upregulated throughout the superficial layers of the SC on the contralateral (injured) side. Here, the dashed lines indicate the boundary of GFAP and iba1 upregulation. Double labelling immunofluorescence reveals that Hsp27 colocalises with the astrocytic marker GFAP (H), but not with the microglial marker iba1 (I), nor with the neuronal marker NeuN (J), nor with the proliferative marker PCNA (K). Scale bar: A–C = 100 µm; D–G = 250 µm; H–K = 50 µm.

The distribution of Hsp27 expression in the SC after ON crush was demarcated more clearly by comparison with the labelling patterns of parvalbumin, calbindin, GFAP and iba1. Parvalbumin immunolabelling in the SC is characterized by a population of small cell bodies that are mainly localised to the stratum griseum superficiale, while calbindin immunolabelling is notable for a band of large cell bodies that are principally localised to the stratum opticum. Double labelling immunofluorescence showed that Hsp27-positive cells were overwhelmingly located in the same strata of the SC as calbindin neurons, namely the stratum opticum (Fig. 9A–C). Upregulation of GFAP and iba1, in contrast, occurred throughout the superficial layers of the SC (Fig. 9D–G). Double labelling immunofluorescence again showed that Hsp27 colocalised with GFAP (Fig. 9H), confirming its presence within astrocytes, but not with the microglial marker iba1, nor with the neuronal marker NeuN, nor with the proliferative marker PCNA (Fig. 9I–K). PCNA-positive cells were present within the superficial layers of the SC, but these cells were microglia not astrocytes (see S3 Fig.).

### Expression of Hsp27 and Hsp70 in the visual cortex during RGC degeneration

**Figure 10:**
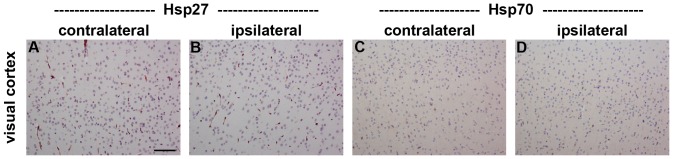
Representative images of Hsp27 and Hsp70 expression in the visual cortex 7d after ON crush. Hsp27 and Hsp70 immunoreactivities are uniformly low throughout the injured (contralateral; A, C) and uninjured (ipsilateral; B, D) visual cortex. Scale bar  = 100 µm.

In the visual cortex of control brains, Hsp27 immunolabelling was limited to some blood vessels, while Hsp70 immunoreactivity was not observed (data not shown). Analysis of the visual cortex of rats subjected to ON crush 3d (data not shown) or 7d previously (Fig. 10) revealed no upregulation of either Hsp in the injured (contralateral) or uninjured (ipsilateral) side.

## Discussion

The current study sought to achieve two objectives. The first aim was to describe the patterns of Hsp27 and −70 expression in the retina in four discrete models of RGC degeneration: axonal injury (ON crush), somato-dendritic injury (NMDA-induced excitotoxicity), chronic hypoperfusion (2VO) and experimental glaucoma. The second aim was to document Hsp27 and −70 expression in the ON/OT and subcortical, retinorecipient areas of the brain during Wallerian degeneration of RGC axons. The results showed that Hsp27 was robustly upregulated in the retina in each injury paradigm, with the chronic models, 2VO and experimental glaucoma, displaying a more persistent Hsp27 transcriptional response than the acute models. Hsp27 expression was always associated with astrocytes and with a subset of RGCs in each of the models excluding NMDA. Hsp27 was present within astrocytes of the ON/OT in control rats. During Wallerian degeneration, Hsp27 was upregulated in the ON/OT and expressed de novo by astrocytes in the LGN and the stratum opticum of the SC. Conversely, the results of our study indicate Hsp70 was not induced in any of the models of injury, either in the retina, or in the ON/OT, or in the subcortical, retinorecipient areas of the brain.

As stated above, we found minimal evidence for induction of Hsp70 by RGCs, or indeed by any other neuronal or glial cell types in the retina, in any of the models of injury. In order to reach this conclusion, we followed a systematic approach involving qPCR, immunohistochemical and Western blotting analyses throughout the time period both prior to, and encompassing, RGC degeneration. The qPCR technique ensured sensitivity to any transcriptional changes, the specificity and effectiveness of the immunohistochemistry methodology was verified by concurrent immunolabelling of brain sections from MCAO rats, and the Western blotting procedure on each retinal sample with Hsp70 yielded a single band of the expected molecular weight and overall densitometry data that corresponded with the qPCR and immunohistochemistry findings. As such, there appears little doubt that Hsp70 is neither acutely, nor persistently, upregulated in situations of RGC injury. One caveat to this conclusion is that we did measure a statistically significant upregulation of Hsp70 mRNA in the 2VO model of injury. The high sensitivity of qPCR likely accounts for the fact that this upregulation was not mirrored by an upregulation in the Hsp70 protein level. We hypothesise that since the 2VO model affects the outer as well as the inner retina, unlike the other three models, that the subtle upregulation of Hsp70 mRNA during 2VO resulted from enhanced constitutive expression by photoreceptors rather than from de novo expression by other retinal cell types.

Our overall finding of minimal upregulation of Hsp70 following RGC injury is in agreement with the results of various studies that assessed Hsp70 expression in paradigms of ischemia [7], 2VO [8] or experimental glaucoma [9], but contradicts the published data of other studies in the retina that positively identified Hsp70 upregulation. How can these disparate results be reconciled? Firstly, the upregulations in retinal Hsp70 expression observed following sublethal hypoxia in cultured RGCs [4], and after ischemia-reperfusion [5] were rapid and transient in nature, occurring up to two hours after the insult. It is quite feasible that similar phenomena were apparent in the current study, but were not detected as the earliest time point analysed was 1d. It can be argued that transient changes are of relatively little relevance in the context of ongoing survival or death of RGCs. Secondly, the upregulations in Hsp70 expression noted in models of NMDA-induced excitotoxicity [11] and experimental glaucoma [10] were also of short durations and characterised by only subtle immunohistochemical changes not verified by Western blotting or other techniques. Taking into account our results and all of the previous body of published data, it can be deduced that the retina does not instigate a robust endogenous Hsp70 response to aid RGC survival.

The situation in the retina contrasts markedly with the brain, where, for example, models of transient and permanent, focal as well as global, ischemia (see [2] for review), excitotoxicity [36], [37], and axonal injury [38] all result in sustained Hsp70 upregulation within neuronal populations. Why does the retina, despite being a part of the central nervous system (CNS), differ so markedly from the brain with respect to its Hsp70 injury profile? The issue is not easily resolved, but two relevant points can be made. Firstly, the retina and brain differ in their expression profile of Hsp70 when healthy, not just as a consequence to injury. Specifically, there is negligible constitutive expression of Hsp70 in the brain. Yet, in the retina, Hsp70 is present within normal photoreceptor cell bodies and inner segments [39]. Photoreceptors are one of the most specialised neurons within the CNS and have extremely high energy demands owing to the continuous synthesis and shedding of disc segments. It has been hypothesised that Hsp70 is persistently induced in photoreceptors to chaperone native proteins against the damaging effects of free radicals produced by light and mitochondrial respiration [2]. Obviously, this does not explain why RGCs themselves do not upregulate Hsp70 after injury, but it does illustrate that the specialised nature of the retina has resulted in the evolution of a differing tissue Hsp profile to the brain. Secondly, the response, or lack thereof, in RGCs can be putatively explained by the fact that these neurons express Hsp27 instead of Hsp70 following induction of damaging stimuli. While Hsp27 upregulation in the brain has been noted in neuronal populations following ischemia [32], this small Hsp is primarily associated with astrocytes rather than neurons. Yet, in the retina, a number of studies [7], [13], [14], [15], [40] have documented robust, persistent expression of Hsp27 by injured RGCs, findings corroborated herein. In this respect, RGCs reflect the functioning of peripheral nerves, where axonal injury leads to induction and prolonged expression of Hsp27, but not of Hsp70 [41], [42]. A similar response is not seemingly intrinsic to central neurons. There is a certain irony here in that the ON is considered a quintessential CNS white matter tract.

Interest in the expression profiles and activities of inducible Hsps is motivated, to a considerable extent, by the ongoing search for factors that prolong RGC survival. Hsp27 [43], [44], [45] and −70 [4], [46], [47], [48] are both firmly implicated as anti-apoptotic, neuroprotective effectors in retinal preconditioning modalities. It is somewhat surprising, therefore, that the effectiveness of neither protein has been investigated using overexpression, transfection or prolonged delivery approaches, as has occurred for neurotrophins, since they represent an appealing strategy for prolonging RGC survival in glaucoma. Our present results show that Hsp27, but not −70, is involved in the endogenous survival response of the retina to RGC injury. It might be argued, therefore, that delivery of Hsp27 would be ineffective in diseases featuring RGC loss, as these neurons already express the molecular chaperone when injured. In fact, only a small proportion of RGCs express Hsp27 during experimental glaucoma and even after ON transection-induced injury, which affects the entire population of RGCs [13]; thus boosting Hsp27 expression may prove an effective tactic.

As highlighted in the introduction, an increased understanding of the pathology of the visual target areas during RGC axonal injury will potentially aid disease diagnosis, provide tissue biomarkers to more precisely assess injury status, and better inform as to suitable neuroprotection strategies. In principal, inducible Hsps represent ideal candidates for each of these goals; however, to date, little concerted information has been available à propos the expression patterns of inducible Hsps in the OT, LGN, SC and visual cortex as Wallerian degeneration of RGC axons progresses: Krueger-Naug and colleagues [13] revealed that Hsp27 is expressed in the OT and SC following transection of the ON, while Zhao et al [49], in a short communication, noted that Hsp70 was associated with cells in the IGL of the LGN. Our results confirm and extend the former data. We have shown that in each of the models of RGC injury, Hsp27 is strongly upregulated in astrocytes within the OT (as well as ON) concurrent with axonal cytoskeleton breakdown. Moreover, Hsp27 was also induced in astrocytes present within the dLGN, IGL, vLGN, and SC. It appears likely that astrocytes in close proximity to degenerating RGC axons and their terminals synthesize Hsp27. We were interested in whether astrocytes within the injured LGN and within the different layers of the SC were quiescent or dividing and how such a response might relate to expression of Hsp27. The results showed that PCNA-positive, proliferative cells were present within both target areas; however, these cells were microglia rather than astrocytes, indicating that astrocytes, while reactive and expressing molecular chaperones such as Hsp27, were not undergoing de-differentiation.

Of particular interest, HSP27-positive astrocytes in the SC were restricted to a narrow band predominantly localised to the stratum opticum. This is an intriguing result as all of the superficial layers of the SC (stratum zonale, stratum griseum superficiale and stratum opticum) receive input from RGC axons, and all three layers display increased astrocytic (GFAP) and microglial (iba1) activity during Wallerian degeneration of RGC axons, as identified herein and by others [17], [50]. Nevertheless, the stratum opticum is the layer most densely innervated by the retina; thus, it is logical that endogenous responses to injury would be more efficacious. Krueger-Naug et al [13] suggested that Hsp27 may be a more precise marker for areas of more severe injury. We would further hypothesize that Hsp27 may represent a useful surrogate marker for assessing the status of LGN and SC injury in short-term RGC neuroprotection studies. To date, the extent of injury and protection in the LGN and SC is dependent upon quantification of neuronal survival, which necessitates experiments of many months [51], [52].

Our study indicates that neither Hsp27 nor Hsp70 are upregulated by neurons in the LGN or SC during RGC axonal degeneration. We paid particular attention to the IGL, which is simple to identify using a combination of parvalbumin and Substance P immunolabelling [53], but we were unable to corroborate the preliminary findings of Zhao et al. [49]. The lack of inducible Hsp27 or −70 expression by target neurons is not unexpected, as transsynaptic neuronal degeneration lags RGC axonal degeneration by a considerable time [51], [52], [54]. A similar comment can be applied to the lack of Hsp27 or −70 expression in the visual cortex. It might be expected that astrocytes in the visual cortex would express Hsp27 following loss of input consequent to RGC degeneration; however, upregulation of Hsp27 presumably only occurs in conjunction with degeneration of the optic radiation, which will arise as transsynaptic neuronal degeneration proceeds in the LGN.

In conclusion, the findings of the present study augment our understanding of the involvement of Hsp27 and Hsp70 in the response of the visual system to RGC degeneration.

## Supporting Information

S1 Fig **Double labelling immunofluorescence of glutamine synthetase with glial markers in the optic chiasm of normal rats.** Upper panel: glutamine synthetase (glut syn)-positive cells colocalise with olig2-labelled oligodendrocytes. Lower panel: in contrast, glut syn-positive cells fail to colocalise with with GFAP-labelled astrocytes. Scale bar  = 50 µm.
(TIF)

S2 Fig **Double labelling immunofluorescence of Hsp27 with glial markers in the dLGN of rats subjected to ON crush one week previously.** Hsp27-positive cells colocalise with GFAP-labelled astrocytes (A–C). Hsp27-positive cells fail to colocalise with the microglial marker iba1 (D–F). Scale bar: A, C, E, G, I, K = 250 µm; B, D, F, H, J, L = 50 µm.
(TIF)

S3 Fig **Double labelling immunofluorescence of PCNA with glial markers in the SC of rats subjected to ON crush one week previously.** Upper panel: PCNA-positive cells fail to colocalise with the GFAP-labelled astrocytes. Lower panel: PCNA-positive cells colocalise with iba1-labelled microglia (D-F, arrows). Scale bar  = 50 µm.
(TIF)

S4 Fig **Underlying data.**
(ZIP)
