# Supplementary figures and images for: Expression of Inducible Heat Shock Proteins Hsp27 and Hsp70 in the Visual Pathway of Rats Subjected to Various Models of Retinal Ganglion Cell Injury
Source: PLoS One. 2014 Dec 23;9(12):e114838. doi: 10.1371/journal.pone.0114838 (PMC4275305; doi:10.1371/journal.pone.0114838)

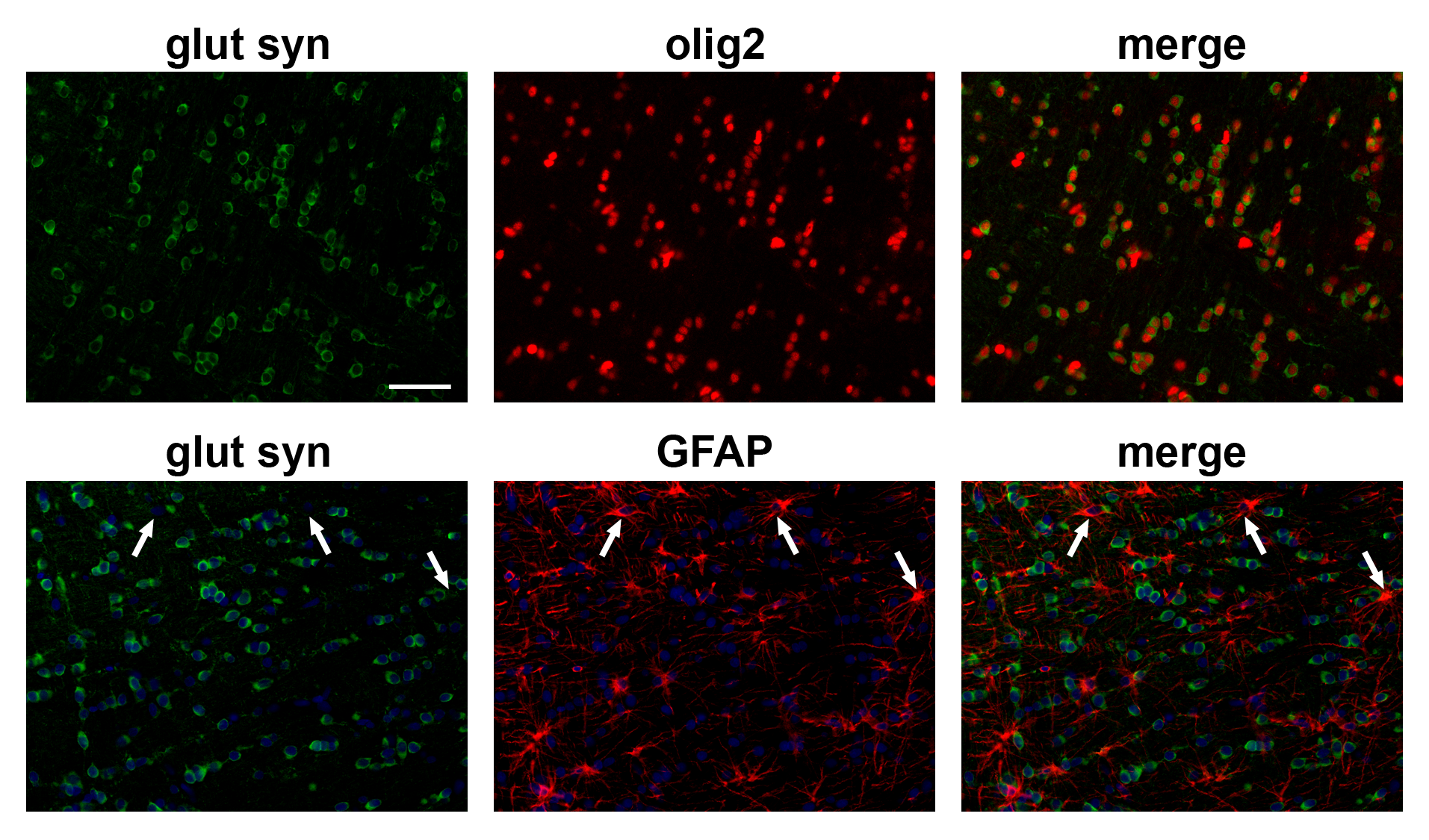

Supplement: S1 Fig — Double labelling immunofluorescence of glutamine synthetase with glial markers in the optic chiasm of normal rats. Upper panel: glutamine synthetase (glut syn)-positive cells colocalise with olig2-labelled oligodendrocytes. Lower panel: in contrast, glut syn-positive cells fail to colocalise with with GFAP-labelled astrocytes. Scale bar = 50 µm. (TIF) [file pone.0114838.s001.tif]

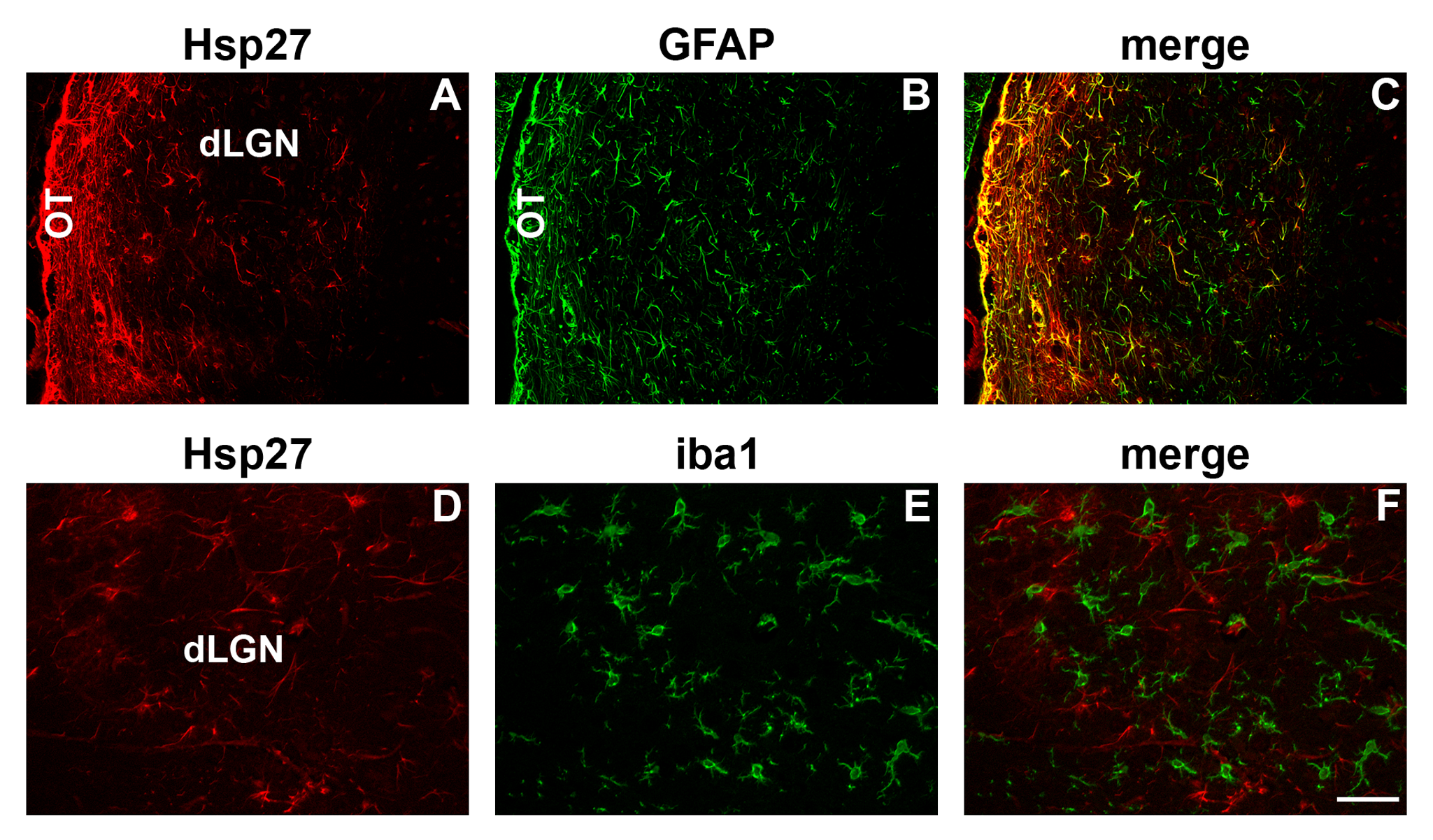

Supplement: S2 Fig — Double labelling immunofluorescence of Hsp27 with glial markers in the dLGN of rats subjected to ON crush one week previously. Hsp27-positive cells colocalise with GFAP-labelled astrocytes (A–C). Hsp27-positive cells fail to colocalise with the microglial marker iba1 (D–F). Scale bar: A, C, E, G, I, K = 250 µm; B, D, F, H, J, L = 50 µm. (TIF) [file pone.0114838.s002.tif]

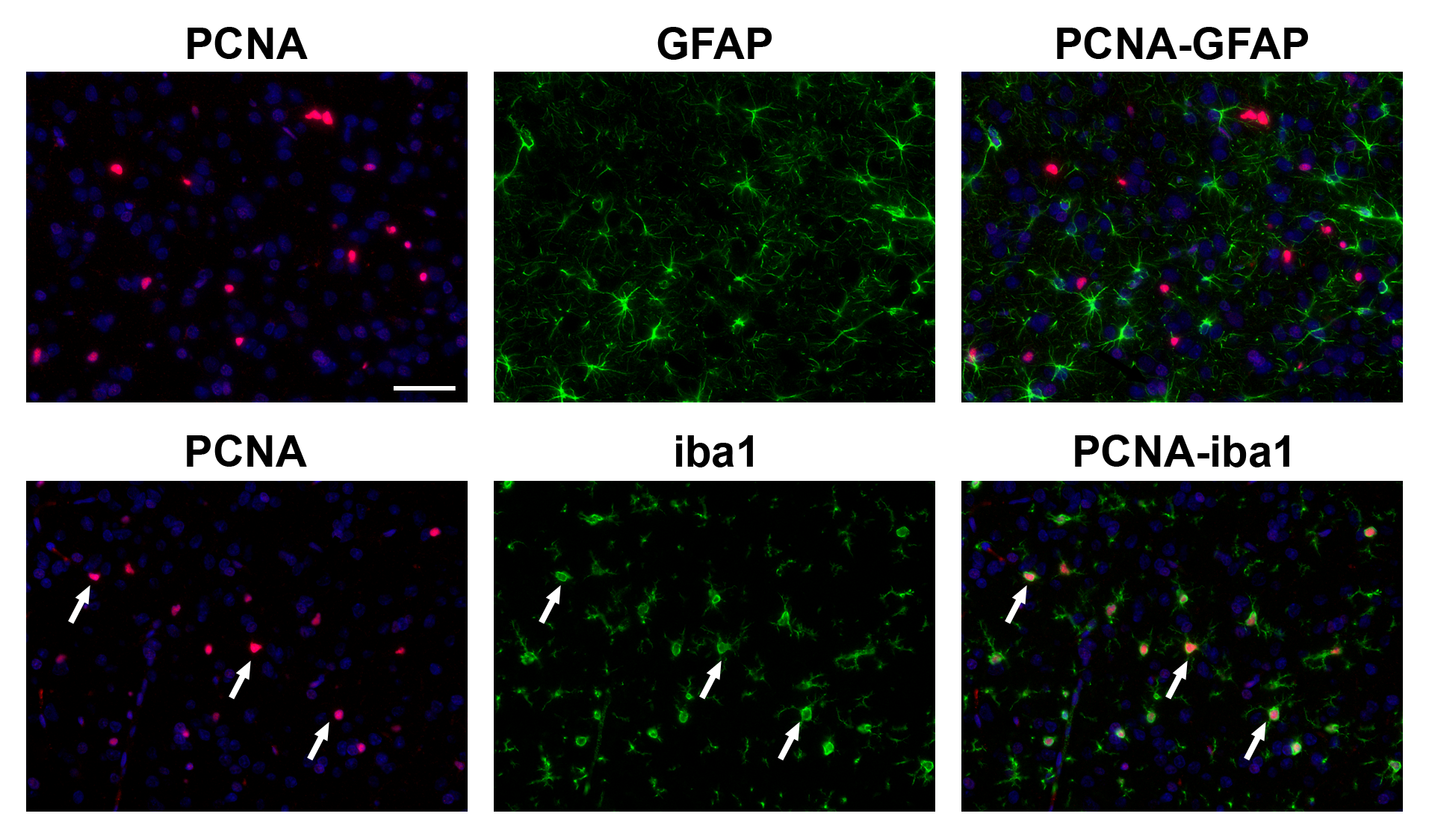

Supplement: S3 Fig — Double labelling immunofluorescence of PCNA with glial markers in the SC of rats subjected to ON crush one week previously. Upper panel: PCNA-positive cells fail to colocalise with the GFAP-labelled astrocytes. Lower panel: PCNA-positive cells colocalise with iba1-labelled microglia (D-F, arrows). Scale bar = 50 µm. (TIF) [file pone.0114838.s003.tif]
